# Surgical management of Hawkins type III talar neck fracture through the approach of medial malleolar osteotomy and mini-plate for fixation

**DOI:** 10.1186/s13018-017-0610-3

**Published:** 2017-07-14

**Authors:** Hui Liu, Zhida Chen, Wenrong Zeng, Yuanfei Xiong, Yongzhi Lin, Huacheng Zhong, Jin Wu

**Affiliations:** 1 Department of Orthopaedics, The Affiliated Southeast Hospital of Xiamen University, Orthopaedic Center of People’s Liberation Army, 363000 Zhangzhou, People’s Republic of China; 2 Department of Medical Imaging, The Affiliated Southeast Hospital of Xiamen University, Orthopaedic Center of People’s Liberation Army, 363000 Zhangzhou, People’s Republic of China

**Keywords:** Hawkins’s classification, Talar neck fracture, Medial malleolar osteotomy, Mini-plate, Anatomical measurement

## Abstract

**Background:**

Fractures of the talar neck are relatively uncommon yet current interventions suffer from a high incidence of complications and poor functional outcomes. In the present study, we report a surgical treatment of Hawkins type III talar neck fracture through the approach of medial malleolar osteotomy and mini-plate for fixation and discuss the therapeutic effects after long-term follow-up.

**Methods:**

From January 2010 to January 2015, 21 patients with 22 fractures were treated using this approach within days of sustaining the injury. Clinical and radiographic data were collected during regular post-operative follow-ups. Health-related quality of life factors were evaluated using visual analogue scale (VAS). Functional outcomes were determined according the Hawkins score and the Ankle-Hind foot Scale of the American Orthopedic Foot and Ankle Society (AOFAS). Present of complications such as arthritis, avascular necrosis (AVN), and malunion were evaluated using radiographs and magnetic resonance imaging (MRI). Anatomical parameters of injured and corresponding uninjured talus were measured and compared using digital three-dimensional (3D) computer model.

**Results:**

The mean duration of surgery was 65.6 ± 9.7 min. The average blood loss volume of the patients was 29.1 ± 5.7 ml. All the patients except 1 were followed up 18 to 41 months (average 29.6 months). The average VAS score for these patients was 3.2 ± 1.1, and the mean Hawkins score was 11.4 ± 3.4 at the final follow-up visit. The average AOFAS score was 72.8 ± 17.3. Nine patients outcomes were rated as “excellent”, 4 as “good”, 4 as “fair,” and 4 as “poor”. No malunion, screw loosening, plate breakage, or other internal fixation failures were found at final follow-up. Long-term complications included: 1 case of malunion, 5 cases of complete AVN, 8 cases of partial AVN, 13 cases of talocrural arthritis, 14 cases of subtalar arthritis, and 3 cases of talonavicular arthritis. Secondary surgery was performed in 4 cases. The relevant average anatomical data of injured and uninjured talus show no significant difference.

**Conclusions:**

This surgical treatment we used here resulted in decreased soft tissue trauma, adequate exposure of talar neck, satisfactory performance of daily life activities, and quality of life following surgery and restoration of anatomy of injured talus. However, long-term complications such as arthritis and AVN are still commonly seen.

## Background

Talar fractures are unusual and potentially debilitating injuries that account for approximately 3 to 6% of foot fractures and less than 1% of all bone fractures [1–3]. Most of these injuries are caused by motor vehicle accidents or falls and are associated with pain, swelling, and bruising of the ankle. Fractures of talus are mainly classified by their location: head, neck, and body [4]. Between 50 and 80% of all talar fractures occur in the neck, whereas only 13 to 23% of talar fractures occur in the body, and only 5 to 10% at the head [4]. In recent years, morbidity of talar fractures has risen with an increase in high-energy accidents and severe falls.

Management and treatment challenges arise for these injuries due to the potential for limited blood supply, multiple articulations, and broad chondral surface area, particularly in complex multiple plane fractures of talus [3, 4]. The Hawkins system is the most widely accepted classification system for talar neck fractures and includes three classifications based on initial fracture displacement: a non-displaced fracture (type I), a displaced fracture where the subtalar joint is subluxated or dislocated (type II), and a dislocation at both the subtalar and tibiotalar joints (type III) [5]. A type IV fracture, which consists of a dislocation at the subtalar, tibiotalar, and talonavicular joint, was subsequently added by Canale and Kelly [6]. A distinction between talar neck fractures and body fractures is not made in this system. In general, any inferior fracture located within or posterior to the talar lateral process is considered a talar body fracture, while a fracture located anterior to the talar lateral process is considered a talar neck fracture [7].

Hawkins type III talar neck fractures indicate injuries to the tibiotalar and subtalar joints [8]. Therefore, the management goal of this fracture is anatomic reduction, which requires attention to proper rotation, length, and angulation of the neck [9]. Various treatment strategies have proven effective for treatment of Hawkins type III talar neck fracture [10–13]. However, these fractures are relatively uncommon and infrequent with much fewer opportunities for surgeons to develop expertise. Thus, poor outcomes and high incidence of complications (i.e., post-traumatic arthritis, avascular necrosis, and malunion) occur frequently. Therefore, this study investigated a surgical treatment strategy of Hawkins type III talar neck fractures through approach of medial malleolar osteotomy and mini-plate for fixation. Therapeutic effects were assessed with an average follow-up time of 29.6 months.

## Methods

### Clinical data

This study is a retrospective analysis of clinical cases. Twenty-one patients (12 males and 9 females) with 22 fractures were enrolled in the study between January 2010 and January 2015. The average age at enrollment was 37.1 years (range 21–53 years). Follow-up was conducted in 21 cases (20 patients) between 18 and 41 months post-operation, with an average of 29.6 months. One patient lost connection at 3-month follow-up. Causes of talar neck fracture included: falls from height in 11 cases (52.4%), traffic accidents in 6 cases (28.6%), and heavy object crushes in 4 cases (19.0%). Three cases were associated with an ankle fracture, 2 cases with contralateral calcaneal fracture, and 1 case with an open fracture. All the patients were diagnosed by clinical symptomatology, X-ray, and 3D computer tomography (CT) and were classified as Hawkins type III according to the Hawkins’s classification.^14^ Study participants were evaluated every 3 months post-operation in the outpatient clinic. Detailed clinical patient parameters are shown in Table 1.

**Table 1:** Detail information of the patients

Variable	Result
Sex	
Male	12 (57.1%)
Female	9 (42.9%)
Age (years)	37.1 (range 21–53 years)
Causes of injuries	
Fall from height	11 (52.4%)
Traffic accidents	6 (28.6%)
Heavy object crushes	4 (19.0%)
Associated injuries	
Ankle fracture	3 (14.3%)
Calcaneal fracture	2 (9.5%)
Open fracture	1 (4.8%)
Follow-up^a^	29.6 (range 18–41 months)

^a^One patient lost connection at 3-months follow-up.

### Pre-operative preparation

Manipulative reductions were performed immediately after the patients were admitted to the hospital and underwent X-ray examinations. Those patients with irreducible dislocations were received emergency surgery. Under general anesthesia with sufficient muscle relaxation, urgent closed reduction was initiated immediately. If the procedure failed, extraarticular facilitated distraction, anterolateral surgical exposure, and Schanz pin-mediated relocation was performed. In one case of open talar fracture where the patient received debridement and suturing in a surgical procedure prior to reduction. Reduction was followed by a short leg plaster boot or calcaneal traction for a period of 3 to 5 days before surgery. The 3D CT examinations were used to visualize the fracture displacement and presence of fragments and small osteochondral fractures for operative plan.

### Surgical procedures

**Fig. 1:**
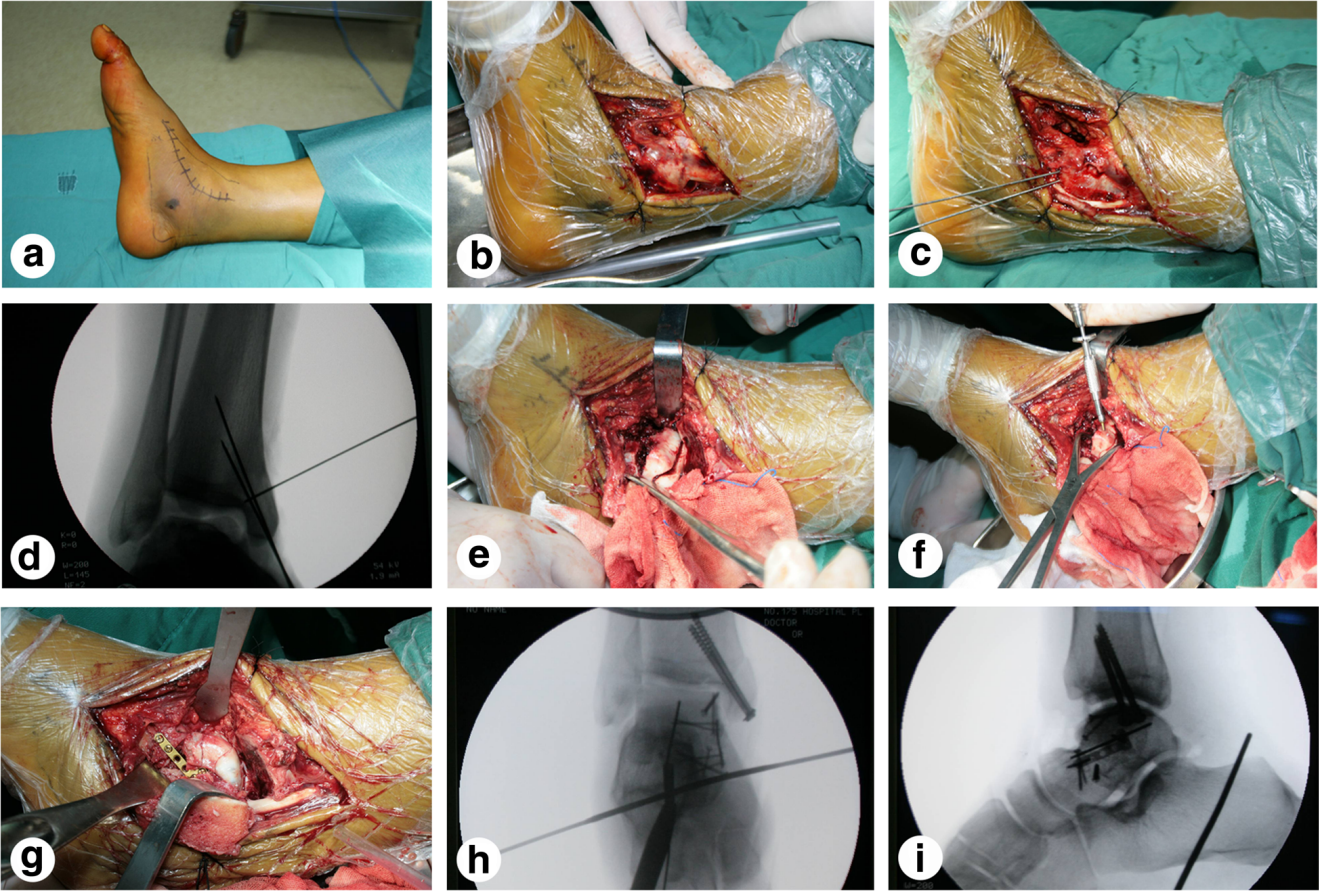
Surgical procedure through approach of medial malleolar osteotomy and mini-plate for fixation (case 3). **a** Arc skin incision. **b** Exposure of malleolus medialis. **c** Preparation for medial malleolar osteotomy. **d** X-ray showing kirschner wire in good position. **e** Medial malleolar osteotomy was performed. **f–g** Talar neck fracture fixed with mini-screws and mini-plate. **h–i** Anterioposterior and lateral view of X-ray showing the screws and plate appropriately fixed

All patients underwent the surgical approach of medial malleolar osteotomy and mini-plate fixation. In brief, the patient was placed in the supine position after continuous epidural anesthesia or general anesthesia. The injured leg was banded with a pneumatic tourniquet and a skin incision approximately 10 cm in length was made between the tibialis anterior muscle and the tibialis posterior tendon along a line from 2 cm proximal to the malleolus medialis to the distal navicular bone (Fig. 1a). The flap was turned up along with the deep fascia to expose the malleolus medialis (Fig. 1b), paying careful attention to protect the saphenous nerve, the great saphenous vein, and the surrounding soft tissue from damage. The location of osteotomy was marked with kirschner wire, and the malleolus medialis was pre-drilled using a high-speed drill (Fig. 1c). Intraoperative radiographs were taken to ensure that the kirschner wire was appropriately fixed (Fig. 1d). The malleolus medialis was then truncated and folded down along with the deltoid ligament (Fig. 1e). To fully explore the articular surface of the tibiotalar joint and the malleolus medialis, the ankle was placed in a varus position. After the fractures were fully exposed, appropriate distraction can be adjusted through the use of the medium-size femoral distractor (Wego Inc., Shandong, China). A 5.0-mm Schanz pin were drilled into the body segment through the fracture for anatomical reduction temporarily. The reduction was verified with intraoperative C-arm fluoroscopy. After the talar neck fracture was reduced, 1.5-mm mini-screws and a 2.0-mm mini-plate (AO, Synthes Inc. West Chester, Pennsylvania, USA) were used to repair the injured chondral surface and for fracture fixation (Fig. 1f, g). Next, the fracture of the malleolus medialis was repaired with two 4.0-mm cannulate lag screws. Intraoperative radiographs were performed to ensure proper placement of screws and the mini-plate (Fig. 1h, i). Finally, the wounds were rinsed and the incisions were closed with a drainage strip at the surgical site.

### Postoperative management

All patients received intravenous antibiotics for 24 h postoperatively. Use of a short leg plaster boot was continued for an average of 4–6 weeks postoperatively. Patients were instructed to practice the digits exercise and were allowed early non-weight-bearing ambulation while wearing the plaster boot. Rehabilitation guidance was provided following the principle of early ambulation and late weight-bearing.

### Outcome assessments

Clinical assessments noted after surgery included: duration of surgery, blood loss volume, VAS score, Hawkins score, and AOFAS hind foot score. Hawkins score consists of three parts: pain, the presence of a limp, and range of motion of the ankle [5]. Pain was assigned from 0 to 6 points: no pain as 6 points, pain only after fatigue as 3 points, and pain after walking as 0 point. The presence of limp ranged from 0 to 3 points: 3 points for no limp and 0 point for the presence of a limp. The ranges of motion of the ankle and subtalar joint were rated from 0 to 3 points, respectively: full motion as 3 points, partial motion as 2 points, and the fixed deformity as 0 point. Overall, “excellent” was classified as a cumulative score of 13 to 15, “good” as 10 to 12, “fair” as 7 to 9, and “poor” as 6 or less. The AOFAS hind foot score was also used to assess functional outcome as described in previous studies [14]. The functional results were obtained regularly postoperatively. Radiological parameters included X-ray at every 3 months post-operation and 3D CT and MRI at every 3 or 6 months post-operation. Radiographic examination analysis evaluated the bony fusion, as well as the occurrence of post-traumatic arthritis, AVN, malunion, and relevant anatomical data of injured and corresponding uninjured talus (Materrialise Mimics 16.0, Belgium).

### Statistical analysis

All anatomical data of injured and corresponding uninjured talus were presented as mean ± standard deviation (SD). Statistical significance was determined by paired *t* test using the SPSS17.0 program (SPSS Inc., Chicago, IL, USA) with *p* < 0.05 being statistically significant.

## Results

### Clinical parameters

Data were expressed as mean ± standard deviation. The mean duration of surgery was 65.6 ± 9.7 min, and the mean intra-operative blood loss volume was 29.1 ± 5.7 ml. At the final follow-up visit, the mean VAS score was 3.2 ± 1.1, and the mean Hawkins score was 11.4 ± 3.4. The functional outcome of 9 (43.0%) cases were classified as “excellent”, 4 (19.0%) cases as “good”, 4 (19.0%) cases as “fair” while the other 4 (19.0%) cases were marked as “poor” at their final follow-up. The average hind foot score of AOFAS was 79.8 ± 17.3 at the final follow-up. In addition, there were two cases of minor surgical complications including one superficial wound infection (4.8%) and one partial wound dehiscence (4.8%). After the dressings were changed, both cases were resolved (Table 2).

**Table 2:** Clinical parameters of cases

Clinical parameters	Result
Duration of surgery (minutes)	65.6 ± 9.7
Blood loss volume (ml)	29.1 ± 5.7
Bony fusion (months)	3.7 ± 1.7
Early complications	
Superficial infections	1 (4.8%)
Partial wound dehiscence	1 (4.8%)
Late complications	
Malunion	1 (4.8%)
Complete necrosis	5 (23.8%)
Partial necrosis	8 (38.1%)
Talocrural arthritis	13 (61.9%)
Subtalar arthritis	14 (66.7%)
Talonavicular arthritis	3 (14.3%)
Secondary procedure	1 (4.8%)
Subtalar arthrodesis	1 (4.8%)
Ankle arthrodesis	1 (4.8%)
Total ankle replacement	1 (4.8%)
Pedicled periosteum flap	1 (4.8%)
VAS score	3.2 ± 1.1
AOFAS hind foot score	79.8 ± 17.3
Hawkins score	11.4 ± 3.4
Excellent	9 (43.0%)
Good	4 (19.0%)
Fair	4 (19.0%)
Poor	4 (19.0%)

### Radiologic assessments

**Fig. 2:**
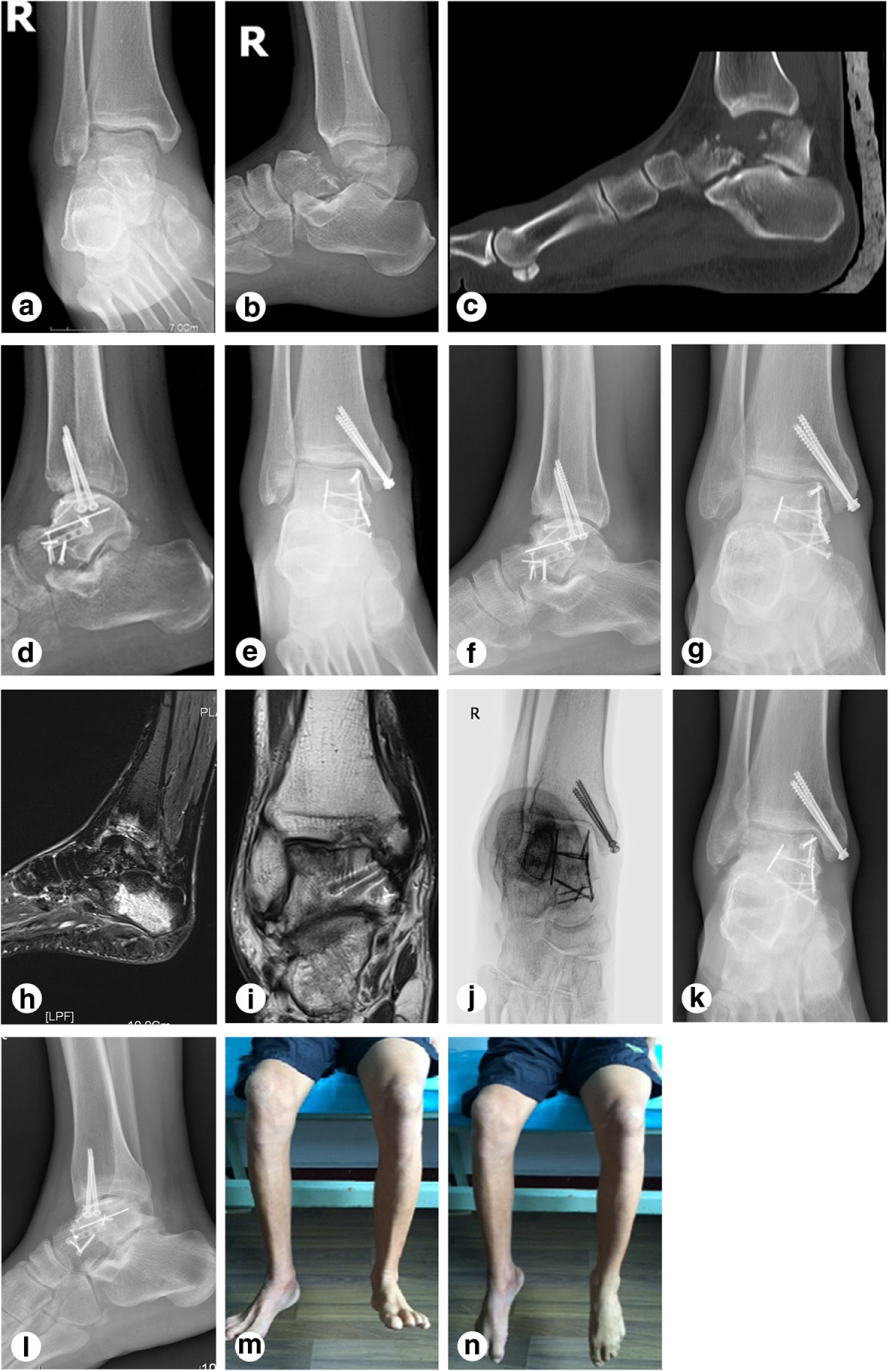
Representative images of case 7 (a 49-year-old male patient with a right ankle injury due to a fall from a 4 m height). **a–c** X-ray, CT, and 3D reconstruction before operation. **d–e** Anterioposterior and lateral view of X-ray at 7 days after operation. **f–g** Anterioposterior and lateral view of X-ray at 6 months follow-up. **h–i** MRI at 2 years follow-up. Talocrural arthritis and partial AVN of the talar bones were observed. **j** pronation/oblique (canale) view of X ray at 3 years follow-up. **k–l** Anterioposterior and lateral view of X-ray at 3 years follow-up. **m–n** Satisfactory range of ankle motion was achieved at 2 years follow-up

**Fig. 3:**
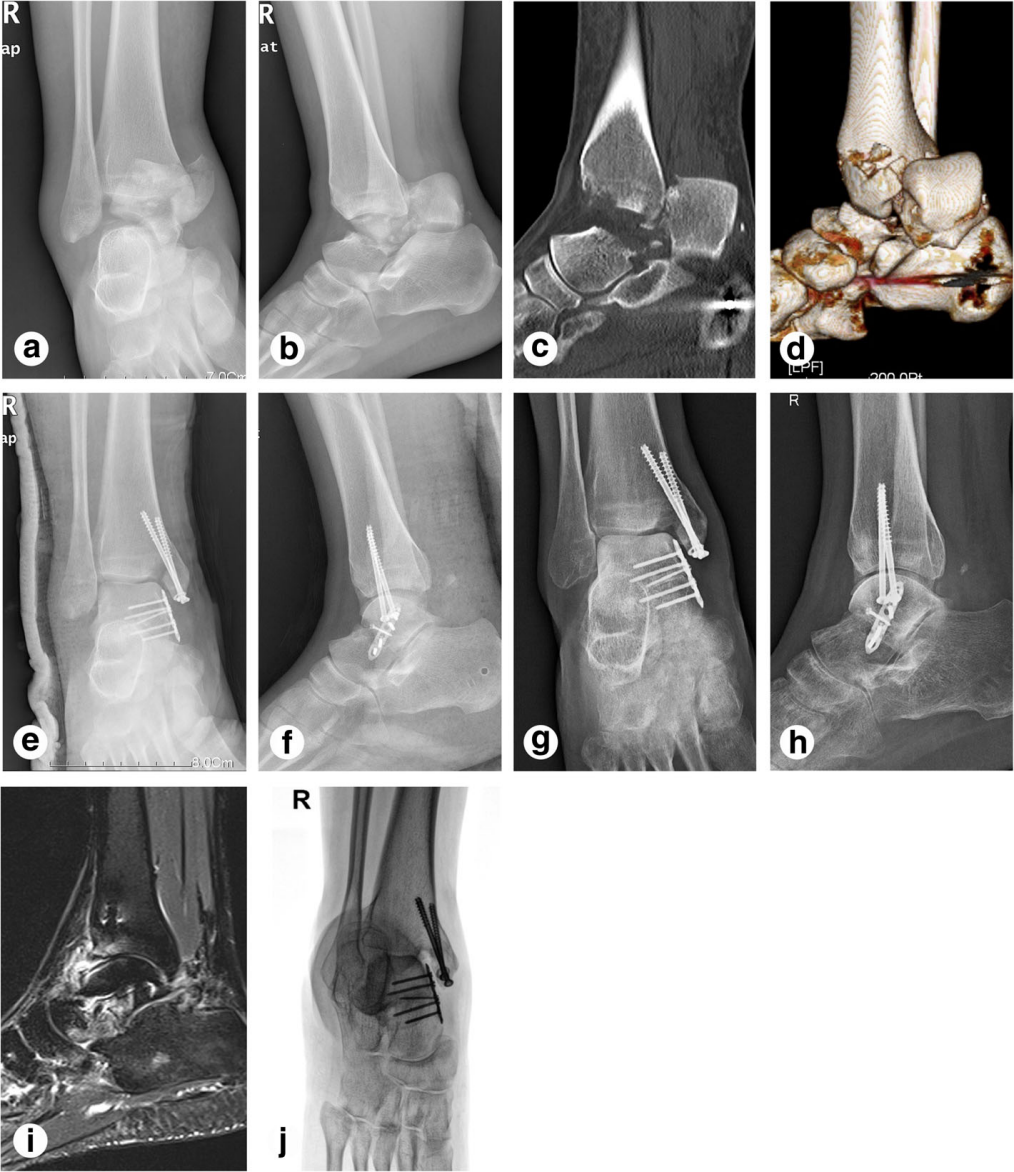
Examples of images obtained from case 18 (A 23-year-old female patient with a right ankle injury due to heavy object crushes). **a–b** Anterioposterior and lateral view of X-ray before operation. **c–d** CT and 3D reconstruction before operation. **e–f** X-ray at 7 days after operation. **g–h** Anterioposterior and lateral view of X-ray at 6 months follow-up. Subtalar arthritis was obviously seen. **i** MRI at 1 year follow-up. Talocrural and subtalar arthritis were obviously seen. **j** Canale view of X ray at 1 year follow-up

**Fig. 4:**
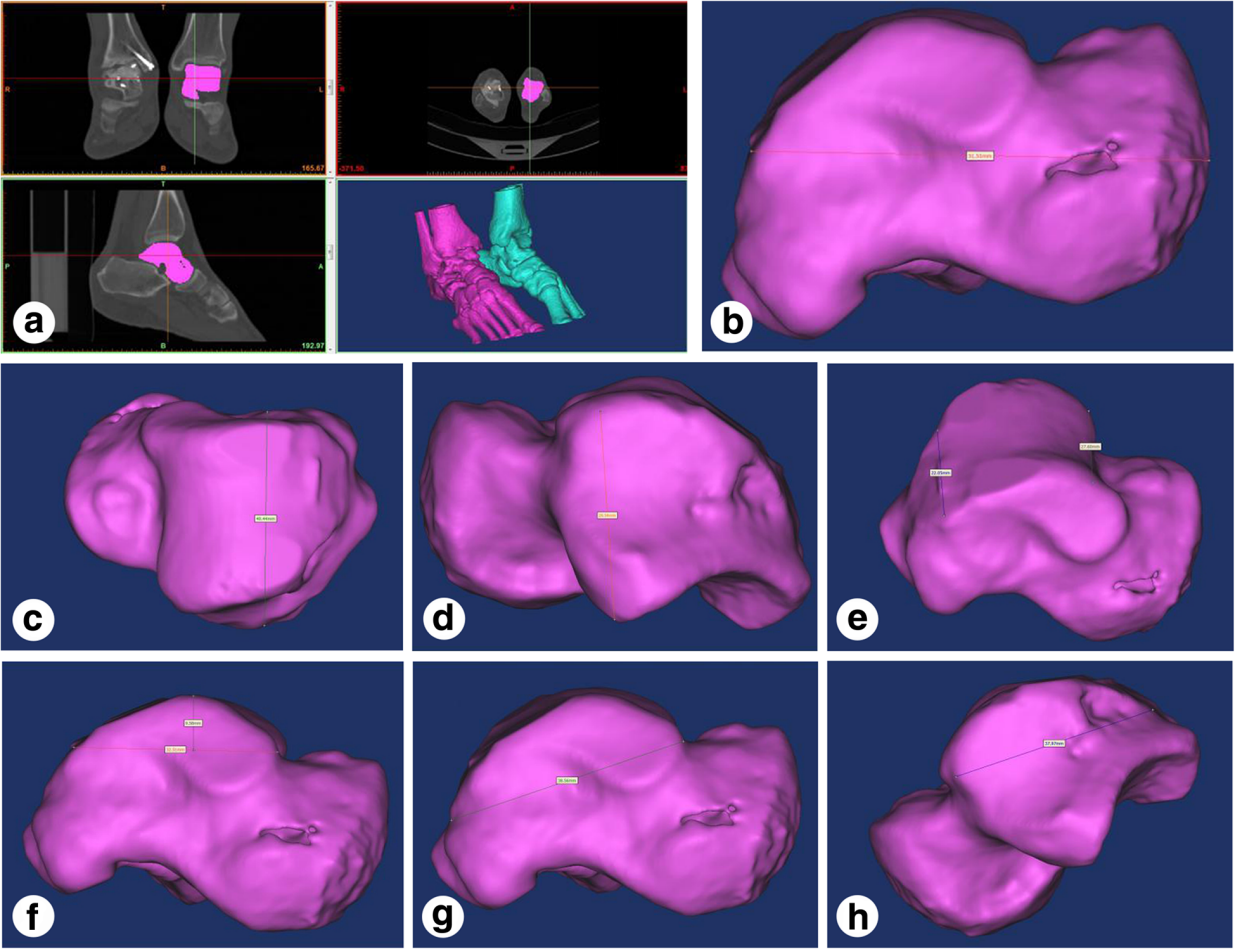
Anatomical measurement of 3D reconstruction of injured and corresponding uninjured talus. **a** Digitalized 3D reconstruction of talus and baseline plane location: transverse section, coronal plane, and sagittal plane. **b** Determination of the length of the talus. **c** Determination of the width of the talus. **d** Determination of the height of the talus. **e** Determination of the anterior and posterior width of the trochlea of talus. **f** Determination of the height of the trochlea of talus and the length of the trochlea of talus. **g** Determination of the length of medial malleolar facet. **h** Determination of the length of lateral malleolar facet

All patients except one achieved successful bony fusion at a mean of 3.7 ± 1.7 months after surgery. Of the 21 cases, 13 (61.9%) cases resulted in AVN, including 5 cases of complete AVN of the talar bones and 8 cases of partial AVN of the talar bones. Post-traumatic arthritis complications were seen in almost all cases: 13 (61.9%) talocrural arthritis cases, 14 (66.7%) subtalar arthritis cases, and 3 (14.3%) talonavicular arthritis cases. Four (19.0%) secondary surgeries included: three cases of arthritis and one case of malunion. In the cases of arthritis, one case received subtalar arthrodesis, 1 case received ankle arthrodesis, and 1 case received total ankle replacement. In the case of malunion, pedicled periosteum flap coverage was performed. No screw loosening, plate breakage, or other internal fixation failures were noted at the final follow-up for any patient. (Table 2). Typical cases are shown in Fig. 2 (case 7) and Fig. 3 (case 18). The digital and virtual reconstruction model of talus and the measurement of anatomical data were shown in Fig. 4. The relevant average anatomical data of injured and corresponding uninjured talus (*n* = 19, except one case lost connection at 3-months follow-up and one case with bilateral fracture) were shown in Table 3, and no significant difference was found.

**Table 3:** The measurement results of the talus ($\overline{x}$ ± s, *n* = 19^a^, mm)

Item	Injured	Uninjured
The length of the talus	50.4 ± 3.6*	51.2 ± 3.8
The width of the talus	41.8 ± 2.9*	41.0 ± 3.1
The height of the talus	29.6 ± 2.7*	30.2 ± 3.3
The anterior width of the trochlea	28.1 ± 2.9*	27.3 ± 2.5
The posterior width of the trochlea	22.9 ± 2.8*	22.6 ± 2.3
The height of the trochlea	9.4 ± 1.6*	9.8 ± 1.2
The length of the trochlea	33.0 ± 3.9*	32.4 ± 2.9
The length of medial malleolar facet	38.1 ± 4.3*	37.6 ± 3.6
The length of lateral malleolar facet	37.9 ± 5.2*	37.1 ± 4.4

*p > 0.05

^a^One patient lost connection at 3-months follow-up, one patient with bilateral talus fracture

## Discussion

### Talus anatomy and fracture characteristics

The talus is a bone located between the fibula, tibia, tarsal navicular, and calcaneus. Two thirds of talar surface is covered with articular cartilage, and there are no muscular origins or insertions into the talus. Blood supply to talus is limited because only the posterior aspect of the talar body and region around the neck exit receive perioster blood supply. The vascularity of the talus consists of three parts: the posterior tibial, anterior tibial, and peroneal arteries [3]. The posterior tibial artery branches out to the deltoid ligament and the artery of the tarsal canal, which is the primary vascular supply to the talar dome; thus, surgeons must aim to protect the deltoid ligament from damage during surgery. Branches of the anterior tibial artery provide the vascular supply for the talar head and the dorsal aspect of the neck, while branches of the peroneal artery contribute to the tarsal sinus and form a vascular sling along with the artery of the tarsal canal. Therefore, the majority of talus fractures result in an AVN due to blood supply interruption. The occurrence of AVN in each Hawkins classification type for displaced talar neck fractures is well described [15, 16]. As previously determined, approximately 80% of AVN cases occur in Hawkins type III or IV talar neck fractures [15], while the risk of AVN is lower for displaced talar body fractures, with an incidence rate of nearly 38% [4].

### Advantages of medial malleolar osteotomy and mini-plate fixation for Hawkins type III talar neck fractures

Hawkins type III talar neck fractures often involve the injuries to the tibiotalar and subtalar joints and are therefore intra-articular fractures [4]. Hence, the surgical management goals of talar neck fractures are fourfold, including anatomical reduction, protection of residual blood supply, and reducing AVN and post-traumatic arthritis. Various surgical strategies have been described for the treatment of the displaced fractures of talus, traditionally consisting of anteromedial, anteriolateral, and medial malleolar osteotomy approaches [8, 13]. However, the anteromedial and anteriolateral approaches require significant dissection and disturbance of soft tissue around the talar head and tarsal sinus that negatively affects the supply blood to talar body. The medial malleolar osteotomy approach not only fully exposes the head, neck, and body of talus but also allows for direct observation of any injuries to the subtalar and tibiotalar joints. Additionally, this approach that cuts midway between the anterior tibial and deltoid artery may better preserve the vascularity of talus [11, 17]. Ziran et al. [18]. and Thordarson et al. [19]. reported that patients with talar fractures receiving the medial malleolar osteotomy approach had satisfactory clinical curative effect postoperatively. Moreover, the mini-plate is a novel internal fixation material more frequently used in recent years as it offers immediate and superior stabilization and fusion [20–22]. Furthermore, biomechanical studies have demonstrated superior rotational resistance and anti-shearing force compared to the absorbable screw, cancellous screw, and cannulate lag screw [21]. In our study, “good” to “excellent” outcomes were obtained in 13 (62.0%) patients evaluated by Hawkins score although long-term complications were still commonly seen. Usually, the anatomical data measurement of talus based on 3D reconstruction had an important impact on the design of surgical instruments and the formulation of individualized treatment programs before operation. In the present work, the relevant average anatomical data of injured and corresponding uninjured talus were measured and compared after operation. The results show the restoration of normal anatomic parameters of injured talus after operation.

## Conclusions

In the present study, Hawkins type III talar neck fractures were treated through the surgical approach of medial malleolar osteotomy and mini-plate fixation. This therapeutic strategy achieves reductions in soft tissue trauma, adequate exposure of talar neck, satisfactory daily life activities and quality of life outcomes, and restoration of normal anatomic parameters of injured talus. We share the experience of our study as follows:

### The advantages of the medial malleolar osteotomy approach and mini-plate fixation

1. The medial malleolar osteotomy approach extends the anteromedial incision to more easily expose the subtalar joints and to achieve anatomical reduction for both coronal and sagittal fractures;
2. Reduction and fixation of the medial malleolus are safer because the location of osteotomy is in non-weight-bearing area;
3. Mini-plate fixation may avoid the compression resulting in talus neck length shortening when applying the cannulate lag screw;
4. Placement of the mini-plate in the medial aspect of talus provided firm axial support for talar head, neck, and body.

### Technical notes for medial malleolar osteotomy and mini-plate fixation in the treatment of Hawkins type III talar neck fractures

1. During surgery, great care must be taken to avoid damage to the deltoid ligament and articular capsule of talus in order to preserve vascularity, particularly when exposing the talus;
2. The surgeon should make an osseous tag at medial malleolar prior to osteotomy and ensure the position of osteotomy is located at the metaphysis;
3. A medium-size femoral distractor could be used to facilitate restoration of tibiocalcaneal height first and then Schanz pin may be used to drill into the body segment through the fracture for anatomical reduction;
4. Rehabilitation guidance provided after surgery should consist of early non-weight-bearing exercises, following the principle of early ambulation, late weight-bearing.

However, long-term complications such as arthritis and AVN are still commonly seen. In addition, prolonged follow-up is necessary to evaluate long-term complications.

## Abbreviations

AVN: Avascular necrosis
CT: Computer tomography
MRI: Magnetic resonance imaging
3D: Three-dimensional
